# Bioinformatics and Structural Characterization of a Hypothetical Protein from *Streptococcus mutans*: Implication of Antibiotic Resistance

**DOI:** 10.1371/journal.pone.0007245

**Published:** 2009-10-02

**Authors:** Jie Nan, Erik Brostromer, Xiang-Yu Liu, Ole Kristensen, Xiao-Dong Su

**Affiliations:** 1 National Laboratory of Protein Engineering and Plant Genetic Engineering, College of Life Sciences, Peking University, Beijing, People's Republic of China; 2 Shenzhen Graduate School of Peking University, Shenzhen, People's Republic of China; 3 University of Copenhagen, Faculty of Pharmaceutical Sciences, Copenhagen, Denmark; University of Washington, United States of America

## Abstract

As an oral bacterial pathogen, *Streptococcus mutans* has been known as the aetiologic agent of human dental caries. Among a total of 1960 identified proteins within the genome of this organism, there are about 500 without any known functions. One of these proteins, SMU.440, has very few homologs in the current protein databases and it does not fall into any protein functional families. Phylogenetic studies showed that SMU.440 is related to a particular ecological niche and conserved specifically in some oral pathogens, due to lateral gene transfer. The co-occurrence of a MarR protein within the same operon among these oral pathogens suggests that SMU.440 may be associated with antibiotic resistance. The structure determination of SMU.440 revealed that it shares the same fold and a similar pocket as polyketide cyclases, which indicated that it is very likely to bind some polyketide-like molecules. From the interlinking structural and bioinformatics studies, we have concluded that SMU.440 could be involved in polyketide-like antibiotic resistance, providing a better understanding of this hypothetical protein. Besides, the combination of multiple methods in this study can be used as a general approach for functional studies of a protein with unknown function.

## Introduction

The Gram-positive oral pathogen *Streptococcus mutans* is the main leading cause of dental caries [1]. As one of the early colonizers, *S. mutans* adheres to the tooth surface and enables the further colonization of other microorganisms, forming dental plaques as a result [2]. Not only enduring a rather acidic environment, these microorganisms also have to withstand various stresses from changes in temperature, nutrition and osmotic pressure variations [3] as well as exposure to natural virulence factors and antibiotics.

For the 1960 ORFs (open reading frames) in the *S. mutans* genome, 63% of them were assigned functions initially through bioinformatics studies and more ORFs, or their orthologs, have been characterized by microarray analysis, phenotype studies and so on [4]–[6]. So far, there are fewer than 500 ORFs of unknown functions (http://cmr.jcvi.org/) [7]. One such case is SMU.440 (GeneID: 1029579), which is composed of 138 residues. There are very few similar proteins in the current databases and these homologs are all hypothetical proteins without known function.

Lateral gene transfer (LGT) [8]–[10] serves as a major way by which organisms acquire novel genes, and it plays an important role in bacterial survival and adaption to environmental changes as well as pathogenity [11], [12]. Thus, studies of LGT can be helpful not only for the understanding of gene evolution and species diversification, but also for the development of drugs that inhibit the transfer of resistance genes. Phylogenetic analysis is a robust method in LGT identification [13]. LGT creates unusually high similarities among organisms, particularly those that are closely related or share the same habitat, which can be used for the detection of LGT [12], [14].

In order to understand the function of unknown ORFs in the *S. mutans* genome, we have initiated a structural genomics project a few years ago in Peking University [15], SMU.440 has been selected as one of the targets. Here, we report the bioinformatics studies and the crystal structure of SMU.440 from *S. mutans*. Phylogenetic analyses suggest that SMU.440 originated via LGT among certain oral pathogens. The crystal structure reveals a fold similar to known polyketide cyclases even though the amino acid sequences are quite different. SMU.440 also shares a similar binding pocket composed primarily of residues with aromatic and acidic side-chains, which points to a potential binding of a polyketide-like molecule.

## Results and Discussion

### Homology Search

**Figure 1:**
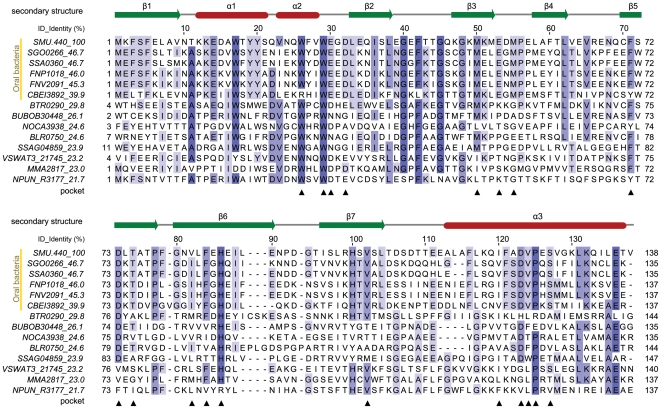
Structure-based multiple sequence alignment of SMU.440 homologs. The corresponding NCBI RefSeq accession numbers and organisms are listed below, SMU.440, NP_720885, *Streptococcus mutans UA159*; SGO0266, YP_001449585, *Streptococcus gordonii str. Challis substr. CH1*; SSA0360, YP_001034364, *Streptococcus sanguinis SK36*; FNP1018, YP_002165260, *Fusobacterium nucleatum subsp. polymorphum ATCC 10953*; FNV2091, ZP_00143517, *Fusobacterium nucleatum subsp. vincentii ATCC 49256*; CBEI3892, YP_001310962, *Clostridium beijerinckii NCIMB 8052*; BTR0290, YP_001608752, *Bartonella tribocorum CIP 105476*; BUBOB30448, ZP_02382082, *Burkholderia ubonensis Bu*; NOCA3938, YP_925122, *Nocardioides sp. JS614*; BLR0750, NP_767390, *Bradyrhizobium japonicum USDA 110*; SSAG04859, YP_002179606, *Streptomyces sp. Mg1*; VSWAT3_21745, ZP_01813727, *Vibrionales bacterium SWAT-3*; MMA2817, YP_001354507, *Janthinobacterium sp. Marseille*; NPUN_R3177, YP_001866578, *Nostoc punctiforme PCC 73102*. The secondary structure was annotated based on the SMU.440 crystal structure. Residues highlighted with colored boxes are conserved to a varying extent, which is illustrated by the darkness of the color. For each protein, name and its sequence identity to SMU.440 are shown in the text columns to the left. Residues forming the cavity are marked by black triangles below the alignment.

SMU.440 is a hypothetical protein without any known functions or protein family classification. A BLAST [16] search against the non-redundant database (NRDB) returns 13 homologs of SMU.440, excluding proteins with short overlaps (<90 residues) or low sequence identity (<20%). The five most similar proteins share more than 40% sequence identities, much higher than the rest of the proteins for which a sudden drop of identities to less than 26% is observed (Fig. 1). Proteins with high identities (>40%) are referred to as SMU.440 close homologs and those with low identities (<26%) are referred to as SMU.440 remote homologs. Among the SMU.440 homologs, SMU.440, SGO0266 and SSA0360 are all proteins from the genus *Streptococcus*. CBEI3892 is from *Clostridium beijerinckii*, which belongs to a very different class from *Bacilli* to which *Streptococcus* belongs. These four genomes are under the same phylum, *Firmicutes*, and they are all from gram-positive bacteria. On the other hand, FNP1018 and FNV2091 are from the genus *Fusobacterium*, in the phylum *Fusobacteria* of gram-negative bacteria. Thus, SMU.440 homologs are sparsely distributed in certain species across broad bacterial domains.

### Phylogenetic Analysis

**Figure 2:**
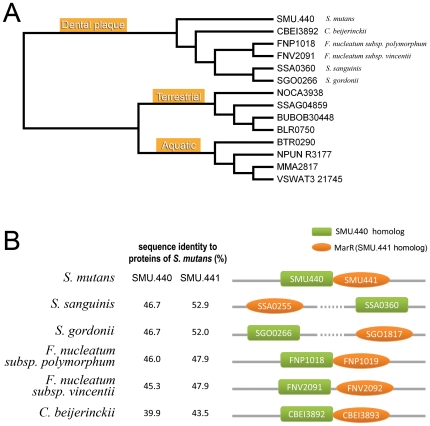
Phylogenetic tree and further analyses of the dental plaque branch. (A) Phylogenetic tree based on SMU.440 homologs. Organism names are indicated for the dental plaque branch in correspondence with the species shown in panel B. (B) Schematic diagram of the loci encoding SMU.440 and MarR (SMU.441) as well as their homologous genes. SMU.440 homologs are shown by green rectangles, and the MarR family proteins are shown by orange ellipses.

A phylogenetic tree was generated based on the amino acid sequences of SMU.440 and its homologs. These 14 proteins were clearly divided into three groups, which are strongly habitat correlated (Fig. 2A). SMU.440 close homologs fell into the same group as SMU.440 and except for the protein from *C. beijerinckii* they are all from organisms known as oral pathogens involved in the formation of dental plaque [17]. Although not considered a typical oral pathogen, *C. beijerinckii* has been isolated from human carious dentin in previous studies [18]. It was earlier shown by Wilson, Kreychman & Gerstein that at levels of sequence identity >40%, precise function is conserved for pairs of single-domain proteins [17]. Thus, SMU.440 and its close homologs are likely to share a similar function.

To search for further proteins with similar function, an iterative search using SMU.440 close homologs was carried out using PSI-BLAST [16] against the NRDB. However, no more sequences were found within the current genome databases, even when lower constrains (overlap>120 aa, identity>30%) were used, which further confirmed the highly specific distribution of SMU.440 with its close homologs in oral pathogen bacteria.

SMU.440 remote homologs are grouped into terrestrial and aquatic bacterial proteins. SMU.440 shares an unusually high similarity to genes from rather divergent organisms. In addition, this scattered phylogenetic distribution appears to be habitat related. Together, it is indicated that LGT has been involved in the spread of SMU.440 close homologs and possibly SMU.440 remote homologs. Besides, most of them are assigned to the polyketide cyclase family (Pfam, PF10604) [19].

### Co-evolution of SMU.440 and SMU.441

In bacteria, proteins with related functions are often clustered into the same operon, which provides useful information for the investigation of proteins with unknown functions. With four overlapping nucleotides in the coding sequences, SMU.440 and the adjacent SMU.441 protein (GeneID: 1027951) are located in the same operon (Fig. 2B). SMU.441 belongs to the MarR protein family of transcription regulators, which is involved in multiple antibiotic resistance [20]. Similar bioinformatics studies were performed on SMU.441. It was found that the top five BLAST hits are from the same organisms as the SMU.440 close homologs (Fig. S1), and their identities form a similar profile as that observed in the SMU.440 BLAST search result (Fig. 2B). Furthermore, if only the N-terminal region (1–40) of SMU.441, which is involved in the dimerization of MarR family proteins and less conserved [20], was selected as a query sequence to search for homologs, only proteins from the five oral bacteria mentioned above were found.

In summary, the homologs of SMU.440 and SMU.441 show a very similar conservation pattern and distribution and their co-occurrences in genomes indicate that the genes encoding these two proteins are related and have been laterally co-transferred at the same time. The splitting of the two genes in some species may be due to later rearrangements of genes after LGT.

### Overall Structure

The crystal structure of SMU.440 has been determined at 2.4 Å resolution using the SIRAS (single isomorphous replacement with anomalous scattering) method and all residues except the last one could be fitted into the electron density. In addition, five residues from the N-terminal His6-tag were also observed in the structure. Statistics from the data collection and structure refinement are summarized in Table 1. The structure has been deposited to the Protein Data Bank and has been assigned PDB ID 3IJT.

**Table 1:** Statistics of X-ray diffraction data, phasing and structure refinement.

Data set	Native	Mercury derivative
Wavelength (Å)	1.095	1.095
Space group	*P*2_1_2_1_2	*P*2_1_2_1_2
Cell dimensions (Å)	a = 93.4, b = 99.7, c = 45.3	a = 93.0, b = 99.9, c = 45.2
Resolution (Å)	34.0–2.38 (2.47–2.38)	34.0–2.40 (2.49–2.40)
Unique reflections	17129 (1662)	15356 (1444)
Multiplicity	2.7 (2.6)	8.6 (8.5)
Completeness (%)	96.9 (97.0)	86.3 (83.4)
<I/σ(I)>	11.94 (2.2)	22.45 (3.91)
*R_sym_* a (%)	3.8 (30.8)	5.6 (41.0)
**Phasing statistics**		
Heavy atom sites		2 x Hg
Resolution (Å)		34–4.0 (34–3.0)
Figure of merit (acentric/centric)		0.36/0.28 (0.18/0.17)
bPhasing power (iso/ano)		0.99/1.27 (0.72/0.91)
c *R_cullis_* (iso/ano)		0.82/0.75 (0.89/0.85)
**Refinement statistics**		
Molecules (ASU)	2	
No. of protein atoms	2267	
No. of waters	90	
Average B-factor (Å^2^) (protein/water)	53.6/53.4	
*R_work_* d (%)	21.1	
*R_free_* d (%)	25.4	
Rms deviation		
Bond length (Å)	0.006	
Bond angle (°)	0.93	
Coordinate error (maximum-likelihood based)	1.61	
Ramachandran plot		
Res. in most favored regions (%)	86.2	
Res. in additional allowed regions (%)	13	
Res. in generously allowed regions (%)	0.8	

Numbers in parenthesis refer to the highest resolution shell (2.47–2.38 Å).

a *R_sym_*  =  ∑*_hkl_*(∑*_i_*[|*I_hkl, i_* − <*I_hkl_*>|]) / ∑*_hkl, i_*<*I_hkl_*>, where *I_hkl, I_* is the intensity of an individual measurement of the reflection with Miller indices *h*, *k*, and *l*, and <*I_hkl_*> is the mean intensity of that reflection.

b Phasing power  =  < phase-integrated lack of closure >/< *F_PH_* – *F_P_*>, where *F_PH_* is the structure factor of the heavy atom derivative and *F_P_* is the structure factor of the native protein.

c *R_cullis_*  =  <[|*F_H, calc_*|/ phase-integrated lack of closure]>, where *F_H, calc_* is the calculated structure factor for the heavy atom.

d *R_work_*  =  ∑*_hkl_*(||*F_o, hkl_*| − |*F_c, hkl_*||) / |*F_o, hkl_*|, where |*F_o, hkl_*| and |*F_c, hkl_*| are the observed and calculated structure factor amplitudes. *R_free_* is defined and calculated in an equivalent manner, but based on a subset of 5% randomly selected reflections.

**Figure 3:**
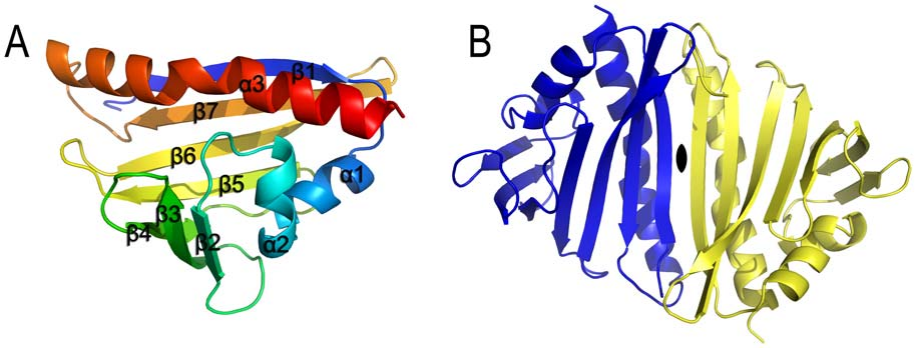
Crystal structure of SMU.440. (A) Overall structure of SMU.440 in cartoon representation. (B) SMU.440 homodimer has a twofold symmetry, the axis of which is indicated in black opal shape.

SMU.440 is comprised of three α-helices and a seven-stranded antiparallel β-sheet, bending into an unclosed β-barrel (Fig. 3A). The structure belongs to the SCOP superfamily of Bet v1-like [21] proteins. There are two molecules per asymmetric unit (ASU) and they form a homodimer via a pair of antiparallel β-strands (Fig. 3B). The dimer has a twofold symmetry, with the twofold axis in the center of the dimer interface and perpendicular to the plane of the extended β-sheet of the dimer. Prediction of assemblies by the PISA server [22] indicates that this dimer interface is the largest (interface area 946.6 Å^2^) with a favorable interaction energy (Δ^i^G −6.9 kcal/mol), in agreement with the dimer state in solution observed during the gel filtration chromatography experiment. However, it is not clear if there is any functional advantage associated with the dimerization.

**Figure 4:**
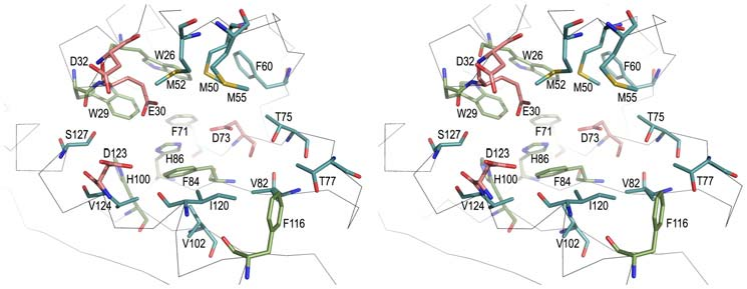
Stereo view of the binding pocket in SMU.440. Residues forming the pocket are shown in sticks; acidic residues are displayed in red, residues containing rings are colored green and neutral residues are shown in blue.

A large forked cavity is formed as the β-barrel wraps around the long C-terminal α3-helix. One end of the cavity is closed by helices α1 and α2 together with the loop between β2 and β3. The volume of the cavity is about 1 050 Å^3^ with a surface area of ∼700 Å^2^. The cavity is comprised of Trp26, Trp29, Glu30, Asp32, Met50, Met52, Met55, Phe60, Phe71, Asp73, Thr75, Thr77, Val82, Phe84, His86, His100, Val102, Phe116, Ile120, Asp123, Val124 and Ser127, most of which are quite conserved residues among the SMU.440 homologs (Fig. 1, Fig. 4). In addition, these residues are spatially distributed in clusters. In the bottom of the pocket are primarily aromatic residues; whereas at the top of the pocket, close to the cavity opening, the residues can be divided into two parts, one side with mainly acidic and the other with neutral residues.

### Comparison to Structures with Known Function

**Figure 5:**
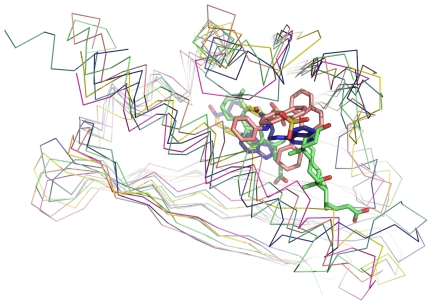
Superimposition of SMU.440 and similar structures with bound ligands. The backbones are shown as ribbons and ligands are shown as sticks. Green, PDB ID, 1fm4; yellow, PDB ID, 2vq5; pink, PDB ID, 1txc; dark blue, PDB ID, 2flh; light blue, PDB ID, 2rer; purple, SMU.440, PDB ID, 3ijt.

A structural similarity search was performed using the DALI web server [23], and the 30 most similar (RMSD <3.0 and Z-score>12) hits all corresponded to polyketide cyclases and class 10 of pathogenesis-related (PR-10) proteins (Fig. 5), if excluding uncharacterized proteins. Polyketide cyclases play an important role in the syntheses of polyketides, where the cyclization patterns diversify the final aromatic products [24]. With a wide distribution throughout the plant kingdom, PR-10 proteins are presumed to be involved in plant resistance in incompatible interactions by binding plant hormones [25]–[27]. Proteins of these two families not only share the same fold, but also have cavities with similar features including the preference for aromatic ligands. SMU.440 does not share a strictly conserved binding site with any protein of these two families. The binding pocket of SMU.440 consists of several conserved aromatic, hydrophobic and acidic residues in orthologous sequences, matching the binding pocket patterns observed in polyketide cyclases and PR10 proteins indicating a potential ability to bind chemically similar classes of ligands. A superimposition of all known ligand complexes of the SMU.440 structural homologs illustrates the common ligand-binding features of this pocket (Fig. 5).

Among the DALI hits, XOXI (PDBID, 3cnw) forms a similar dimer interface as that of SMU.440. Being a hypothetical protein from *Bacillus cereus*, XOXI is predicted to belong to polyketide cyclase family by Pfam. Furthermore, a profile-profile alignment was performed using the FFAS03 server [28], which showed that SMU.440 was more similar to the polyketide cyclase family proteins (Pfam, PF010604; score, −44) than to the PR-10 family proteins (Pfam, PF00407; score, −24) in sequences. This corresponds to the previous observation that SMU.440 remote homologs belong to the polyketide cyclase family.

In conclusion, we have observed that SMU.440 is only found and conserved in a small number of dental pathogenic bacteria through sequence and phylogenetic analysis. Further analysis shows that SMU.441, a MarR protein from the same operon as SMU.440, shares the same distribution pattern and very similar level of sequence conservation. It is thus suggested that SMU.440 has co-evolved with SMU.441, and that SMU.440 may be involved in antibiotic resistance. The determination of the SMU.440 crystal structure revealed a cavity which is similar to that of hormone and especially polyketide binding proteins sharing the same fold, indicating a polyketide-like binding site in SMU.440 homologs. The results reported here shed light on a likely function of a hypothetical protein found exclusively in the dental habitat bacteria.

## Materials and Methods

### Bioinformatics Analysis

BLAST [16] was used for homology search against the NRDB. The output sequences were input into the software MUSCLE [29] and a phylogenetic tree was generated based on the alignment. The programs JALVIEW2.4 [30] and TREEVIEW [31] were used for alignment analysis and tree visualization, respectively. The cavity in the structure was analyzed using the web server CASTp [32] and DALI [33] was used to identify structures that share similarity with SMU.440.

### Protein Expression and Purification

The SMU.440 gene was amplified from genomic *S. mutans* DNA by PCR using the primers SMU.440-F 5′–GCGGATCCATGAAATTTTCTTTTGAATTGG-3′ and SMU.440-R 5′-CCGCTCGAGTCATACTGTCTCCAAGATTT-3′, which contain Bam HI and XhoI restriction sites, respectively. After digestion with BamHI and XhoI, the PCR amplified fragment was ligated to the pET-28a (+) expression vector (Novagen, USA), which was linearized with the same two restriction enzymes. Recombinant clones were selected and sequenced for verification. *E. coli* BL21 (DE3) cells transformed with plasmids encoding SMU.440 were grown in Luria–Bertani broth supplemented with 50 µg ml^−1^ kanamycin at 310 K until the optical density at 600 nm reached 0.6. Recombinant protein expression was induced by adding isopropyl-β-d-thiogalactopyranoside to a final concentration of 1.0 mM, after which the culture was incubated for 4 hours at 303 K. Cells were harvested by centrifugation at 6 700 *g* for 10 minutes at 277 K. The cell pellet was re-suspended in lysis buffer (20 mM Tris-HCl pH 7.5, 500 mM NaCl) supplemented with 1 mM phenylmethylsulfonyl fluoride, and then lysed by sonication. The crude cell extract was clarified by centrifugation (30 000 *g* for 1 hour at 277 K) and the supernatant was purified using a Ni^2+^ chelating column (GE Healthcare, USA). The protein was eluted with a buffer containing 20 mM Tris-HCl pH 7.5, 500 mM NaCl, 500 mM imidazole and further purified by size exclusion chromatography on a HiLoad Superdex 75 column (GE Healthcare, USA) using an elution buffer containing 20 mM Tris-HCl pH 7.5, and 150 mM NaCl. The protein was concentrated to 10 mg ml^−1^ using an Amicon Ultra-15 concentrator (Millipore, USA). The purity of the SMU.440 protein was about 95% as judged by SDS-PAGE analysis.

### Crystallization

Crystallization trials were performed by the hanging-drop vapor-diffusion method at 289 K using 24-well VDX plates (Hampton Research, USA). Crystallization drops were prepared by mixing 1 µl protein with 1 µl reservoir solution, followed by incubation at 289 K. Crystals were observed in several conditions tested in our initial experiments using the crystallization screening kits Crystal Screen, Crystal Screen II and Index Screen (Hampton Research, CA, USA). After optimization, well diffracting crystals were obtained using a reservoir solution containing 0.2 M (NH_4_)_2_SO_4_, 0.1 M Tris-HCl pH 7.0 and 25% (w/v) PEG 3350. Mercury derivatives were prepared by soaking the crystals in the same solution supplemented with 2.0 mM ethylmercury thiosalicylate for three hours.

### Data Collection and Processing

Diffraction data were collected on the I-711 beamline at MAX-Lab (Lund, Sweden) equipped with an Oxford Cryosystem and a Mar165 CCD detector. Crystals were flash cooled without further cryo-protection in a nitrogen cryostream. Data were collected at 100 K and indexed, integrated and scaled using DENZO and SCALEPACK from the HKL package [34] (Table 1). POLARRFN [35] was used to calculate the self-rotation function, which revealed the presence of strong twofold non-crystallographic symmetry (NCS). The crystals contain two molecules per ASU with an estimated Matthews coefficient [36] of 3.36 Å^3^Da^−1^ and a solvent content of 63%.

### Phasing and Model Building

The software SOLVE [37] was used to search for heavy atoms and two mercury atoms were located per ASU. With both the native and the mercury derivative data, SIRAS phases were calculated using SOLVE and improved by solvent flattening using the program DM. Automatic model building was carried out with the software RESOLVE [37] and 112 residues were traced per ASU. In the partially built model, two helices could be assigned to each of the two molecules per ASU. A least square (LSQ) matching of the two helices was performed using the LSQ function in the program O [38] and the twofold NCS axis was located. The initial phases were improved using the program SHARP [39] (Table 1) and by density modification using DM [40]. As a result of imposing NCS averaging and using the automated tracing in RESOLVE, 218 residues were traced. The programs O and CNS were used for manual model building of the remaining parts and PHENIX.refine [41], [42] for the final crystallographic refinement. Structure figures were generated with the software PYMOL [43].

## Supporting Information

Figure S1 Multiple sequence alignment of SMU.441 homologs. The corresponding NCBI RefSeq accession numbers and organisms are listed below, SMU.441, NP_720886, *Streptococcus mutans UA159*; SGO1817, YP_001451086, *Streptococcus gordonii str. Challis substr. CH1*; SSA0255, YP_001034264, *Streptococcus sanguinis SK36*; FNP1019, YP_002165261, *Fusobacterium nucleatum subsp. polymorphum ATCC 10953*; FNV2092, ZP_00143518, *Fusobacterium nucleatum subsp. vincentii ATCC 49256*; CBEI3893, YP_001310963, *Clostridium beijerinckii NCIMB 8052*. Residues highlighted with colored boxes are conserved to a varying extent, which is illustrated by the darkness of the color. For each protein, name and its sequence identity to SMU.441 are shown in the text columns to the left.
(2.57 MB TIF)
