# Supplementary figures and images for: Bioinformatics and Structural Characterization of a Hypothetical Protein from Streptococcus mutans: Implication of Antibiotic Resistance
Source: PLoS One. 2009 Oct 2;4(10):e7245. doi: 10.1371/journal.pone.0007245 (PMC2749211; doi:10.1371/journal.pone.0007245)

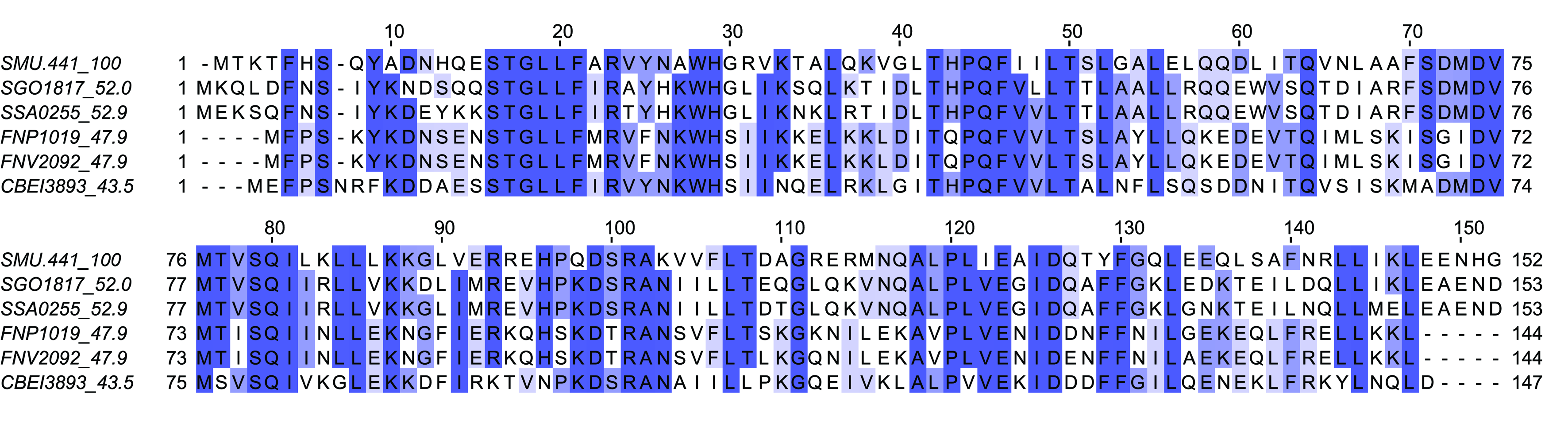

Supplement: Figure S1 — Multiple sequence alignment of SMU.441 homologs. The corresponding NCBI RefSeq accession numbers and organisms are listed below, SMU.441, NP_720886, Streptococcus mutans UA159; SGO1817, YP_001451086, Streptococcus gordonii str. Challis substr. CH1; SSA0255, YP_001034264, Streptococcus sanguinis SK36; FNP1019, YP_002165261, Fusobacterium nucleatum subsp. polymorphum ATCC 10953; FNV2092, ZP_00143518, Fusobacterium nucleatum subsp. vincentii ATCC 49256; CBEI3893, YP_001310963, Clostridium beijerinckii NCIMB 8052. Residues highlighted with colored boxes are conserved to a varying extent, which is illustrated by the darkness of the color. For each protein, name and its sequence identity to SMU.441 are shown in the text columns to the left. (2.57 MB TIF) [file pone.0007245.s001.tif]
